# The Anticonvulsant Effects of Baldrinal on Pilocarpine-Induced convulsion in Adult Male Mice

**DOI:** 10.3390/molecules24081617

**Published:** 2019-04-24

**Authors:** Xiao Zhang, Xing Li, Ning Liu, Ping Zheng, Lin Ma, Fengying Guo, Tao Sun, Ru Zhou, Jianqiang Yu

**Affiliations:** 1 Department of Pharmacology, Ningxia Medical University, Yinchuan 750004, China; zhangxiao5922017@126.com (X.Z.); lixing5922017@126.com (X.L.); liuning234@126.com (N.L.); zhengping122@126.com (P.Z.); 2 Ningxia Key Laboratory of Craniocerebral Diseases of Ningxia Hui Autonomous Region, Ningxia Medical University, Yinchuan 750004, China; linma492201@126.com (L.M.); suntao@126.com (T.S.); 3 College of Basic Medicine, Ningxia Medical University, Yinchuan 750004, China; 19651210@163.com; 4 Ningxia Hui Medicine Modern Engineering Research Center and Collaborative Innovation Center, Ningxia Medical University, Yinchuan 750004, China

**Keywords:** anticonvulsant, Baldrinal, pilocarpine, neuroprotective, neurotransmitter, γ-aminobutyric acid, astrocytes

## Abstract

Epilepsy is a prevalent neurological disorder that was reported to affect about 56 million people in the world. Approximately one-third of the epileptic patients that suffer from seizures do not receive effective medical treatment. The aim of this study was to determine the potential anticonvulsant activities of Baldrinal (BAL) with a mouse model of pilocarpine (PILO)-induced epilepsy. The mice were treated with different doses of BAL or sodium valproate prior to PILO injection. Spontaneous and evoked seizures were evaluated from EEG recordings, and their severity was tested by the Racine scale. In addition, the brain tissues were analyzed for histological changes, and the in situ levels of glutamic acid (Glu) and gamma-aminobutyric acid (GABA) were also measured. Activation of astrocytes in the hippocampus was measured. PILO-treated mice showed a significant increase in Glu levels, which was restored by BAL. In addition, BAL treatment also reduced the rate of seizures in the epileptic mice, and ameliorated the increased levels of NMDAR_1_, BDNF, IL-1β and TNF-α. Taken together, BAL has a potential antiepileptic effect, which may be mediated by reducing the inflammatory response in the PILO-induced brain and restoring the balance of GABAergic and glutamatergic neurons.

## 1. Introduction

In 2015, it was reported that about 65 million people worldwide suffered from epilepsy, and nearly 80% of the patients were from developing countries. Epilepsy-related deaths increased from 112,000 in 1990 to 125,000 in 2015. Epilepsy is characterized by recurrent seizures [1,2] that could lead to behavioral anomalies, such as depression, anxiety, psychosis, and cognitive deficits, all of which affect the quality of life of the patients and their families [3]. More than 60% of the patients suffering from recurrent seizures are prescribed anti-epileptic drugs, which unfortunately have considerable side effects like ataxia and cognitive deficit [4], thereby limiting their preventive usage. Therefore, novel anti-seizure drugs with minimal side effects must be developed [5,6,7].

**Figure 1:**
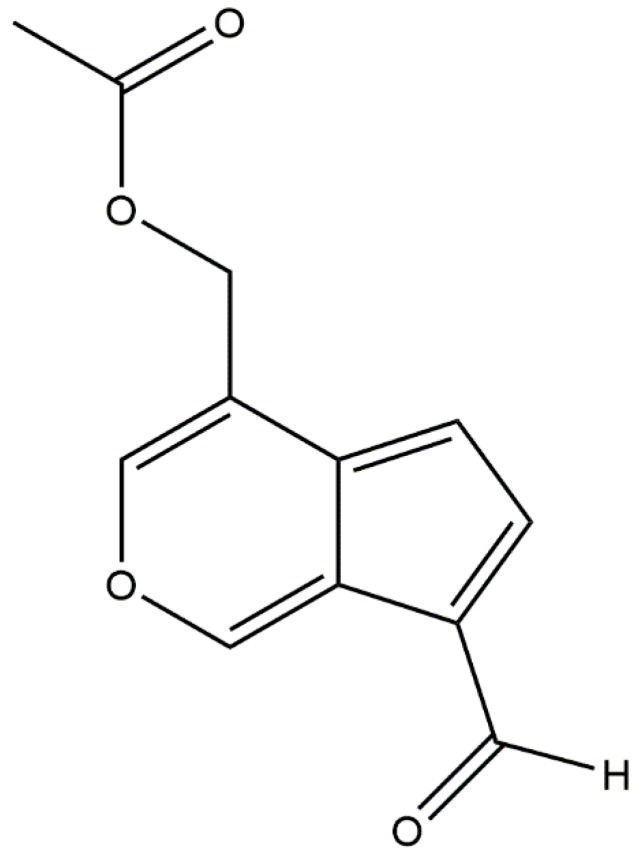
Structure of Baldrinal (BAL).

Plant-derived extracts or compounds have been used for thousands of years in folk medicine for treating epilepsy and have attracted considerable attention in recent years [8,9]. Valerian has long been used to treat insomnia, mood disorders, anxiety, menstrual cramps and psychological stress. It is classified as a dietary supplement under the Dietary Supplement Health and Education Act of 1994 and is an effective sleep aid [8]. Baldrinal (BAL, structure shown in Figure 1) is derived from the extracts of valerian rhizomes and roots. In addition, a high dose of BAL effectively reduces locomotor activity and promotes sleep in mice, indicating its ability to suppress excitatory neural transmission [8,10].

For a long time, epilepsy has been considered to be caused by dysfunctional neurons. Hence, searching for new antiepileptic drugs has focused largely on compounds that affect neuronal function. As efficacy and tolerability of these drugs have not substantially improved over the past decades, and all known antiepileptic drugs merely suppress symptoms without treating the underlying disorder, new strategies in antiepileptic drug development are required [11,12]. In this context, glial cells and astrocytes have been received increasing attention. These cells play essential roles in brain physiology: they modulate synaptic transmission and control ion homeostasis and blood–brain barrier integrity [13,14]. Impairment of these functions has been associated with the pathophysiology of neurological disorders including epilepsy, yet the underlying mechanisms remain enigmatic [15,16]. Abnormal astrocyte, including chronically activated astrocytes and microglia, glial scars are a prominent feature of epileptic foci in the human brain and in experimental epilepsy models [15,16]. The major mechanisms of seizures and epilepsy development facilitated by astrocyte mainly include increasing neuron excitability and inflammation [17]. First, epilepsy disrupts brain homeostasis and leads astrocyte to release Glu [18,19], which disrupts the balance between excitatory and inhibitory neurotransmitters. In addition, the take-up of glutamic acid by astrocytes is also affected. Under normal conditions, part of the glumatic acid released by the presynaptic membrane is taken-up by glutamate transporters of astrocytes. In temporal lobe epilepsy (TLE), decreased expression of the astrocyte glutamate transporters were found [20]. Further, in the epileptic brain, astrocytes undergo significant changes in their morphology and proliferation, which can release an array of inflammatory cytokines, including interleukin (IL)-1β, IL-6, and tumor necrosis factor-α (TNF-α) [21,22]. These pro-inflammatory cytokines can aggravate astrogliosis and enhance the epileptogenic inflammatory signaling via the activation of specific receptors [23]. In this vicious circle, these pro-inflammatory cytokines rapidly accumulate and influence the synaptic transmission, which further influences neuronal excitability and contributes to seizure generation [22] and neuronal damage [20,23]. All in all, activated astrocytes lead to increased inflammation and excitability, and the release of inflammatory cytokines further aggravates the imbalance between excitability and inhibition [24].

These important preclinical studies provide new insights into the regulation of inflammation in the epileptic brain and guide drug discovery to develop promising strategies to improve the therapeutic potential for seizures and epilepsy. BAL has been shown to inhibit autonomic activity in previous studies [8,9]. Most of the sedative-hypnotic drugs work through increasing neuronal inhibition. Besides, a series of BAL compounds have anti-inflammatory effects [25]. Therefore, we hypothesized that BAL might play an antiepileptic role by modulating astrocyte activation, inhibiting the inflammation and decreasing neuronal excitability. Therefore, pilocarpine (PILO) epilepsy model was used to detect the inflammatory cytokines in the epileptic brain and the function of GABAergic as well as glutamatergic neurotransmission in order to find the BAL possible antiepileptic mechanism.

## 2. Results

### 2.1. BAL Attenuates the Behavior and EEG Patterns of Epileptic Mice

According to the Racine scale, the PILO mice exhibited a high seizure score (4–5), characterized by generalized tonic, rearing, convulsion with status epilepticus (SE), and even death. In contrast, no signs of seizure activity were observed in the controls and BAL + PILO mice, while the VPA + PILO mice exhibited low seizure score (1–3), characterized by scratching, head bobbing and forelimb clonus. Compare to PILO group, latency to the first convulsion was significantly delayed when pre-treated with 50 and 100 mg/kg BAL, respectively. However, low BAL dose of 25 mg/kg did not significantly alter the latency period. The incidence of SE was significantly decreased when the mice were pre-treated with a high dose of BAL (50 mg/kg, 100 mg/kg). The survival rate was 100% in the controls (CON), BAL 100 and VPA-treated groups. Compared to PILO group, the survival rate was significantly increased when pre-treated with 50 and 100 mg/kg BAL, respectively. (Table 1)

**Figure 2:**
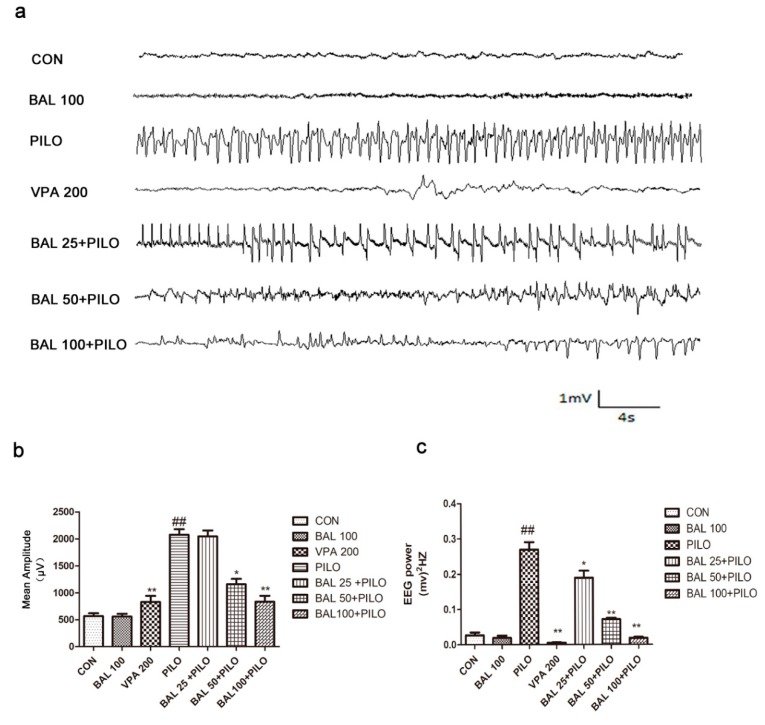
Electroencephalogram (EEG) recordings after PILO treatment. Representative EEG recordings 25 min post PILO injection (**a**). The mean amplitude of seizure EEG (**b**). The total EEG power of seizure (**c**). Results are expressed as mean ± SEM (*n* = 12). ^##^ *p* < 0.01 (Student-Newman-Keuls) compared to the CON group, * *p* < 0.05 (Student-Newman-Keuls) compared to the PILO group, and ** *p* < 0.01 (Student-Newman-Keuls) compared to the PILO group (One-way ANOVA and the Student-Newman-Keuls).

EEG (electroencephalogram) was monitored for 60 min after PILO administration to confirm seizures. In the absence of any pre-treatment, PILO injection resulted in seizure activities, such as a gradual increase in the amplitude with spike and spike-wave complexes and frequent poly spikes. In contrast, pre-treatment with 50 and 100 mg/kg BAL significantly reduced the mean amplitude (*p* < 0.05 and *p* < 0.01) and the total power (*p* < 0.05 and *p* < 0.01), whereas BAL 25 had no significant effects. Taken together, high doses of BAL or VPA attenuated the PILO-induced behavioral changes as well as the electrographic severity of the PILO-induced seizures (Figure 2).

### 2.2. BAL Restores Glutamic Acid and GABA Levels in the Brain of Epileptic Mice

**Figure 3:**
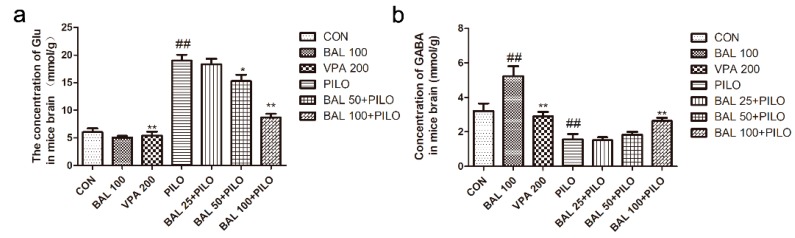
After the PILO injection for 72 h the concentration of GABA and glutamic acid in the brain. glutamic acid concentration(**a**), GABA concentration(**b**). Data are expressed as mean ± SEM (*n* = 6), ^##^ *p* < 0.05 (Student-Newman-Keuls) compared to CON group, * *p* < 0.05 (Student-Newman-Keuls) compared to PILO group and ** *p* < 0.01 (Student-Newman-Keuls) compared to the PILO group (One-way ANOVA and the Student-Newman-Keuls).

Glu levels in the brain significantly increased after PILO administration (*p* < 0.05), and decreased with BAL high dose (100 mg/kg). In contrast, GABA levels were significantly decreased after PILO administration (*p* < 0.05), and restored after pre-treatment with 100 mg/kg BAL (*p* < 0.01, Figure 3). Interestingly, high-dose BAL treatment of the non-epileptic mice did not significantly affect glutamic acid levels but increased that of GABA compared to the controls (*p* < 0.01).

### 2.3. BAL Alleviates PILO-Induced Neurodegeneration

**Figure 4:**
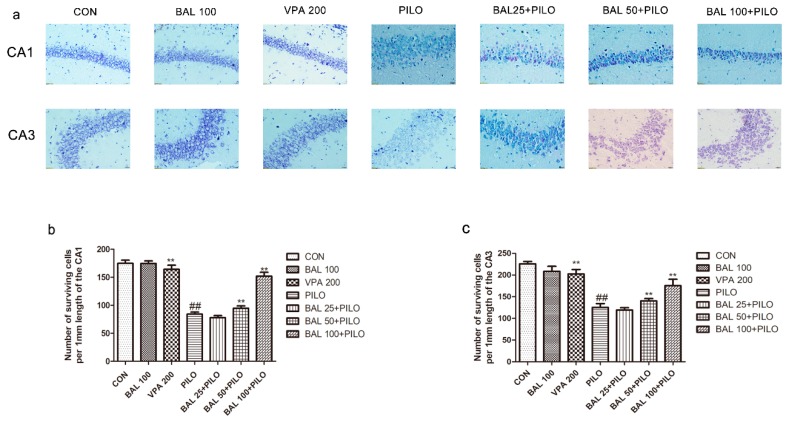
Nissl staining of the hippocampal CA_1_ and CA_3_ pyramidal neurons with cresyl violet 72 h after convulsion (×400 magnification). Nissl-positive cells in CA_1_, CA_3_ are shown for (**a**). Bar 20 μm. The number of surviving neurons in the hippocampal CA_1_ (**b**) and CA_3_ (**c**). Results are expressed as mean ± SEM (*n* = 6). ^##^ *p* < 0.05 (Student-Newman-Keuls) compared to control group, * *p* < 0.05 (Student-Newman-Keuls) compared to PILO group, and ** *p* < 0.01 (Student-Newman-Keuls) compared to the PILO group (One-way ANOVA and the Student-Newman-Keuls).

Nissl staining showed a large number of pyramid-shaped neurons with dense cytoplasmic granules and Nissl bodies in the hippocampal CA_1_ and CA_3_ regions of the CON mice. 72 h after PILO administration, most neurons disappeared, and the remaining cells were irregular, swollen and disintegrating in PILO group. In addition, the number of the Nissl bodies was significantly reduced, and nuclear pyknosis was also detected. Pre-treatment with BAL alleviated neuronal degeneration in the CA_1_ and CA_3_ regions of the hippocampus. The number of surviving cells were counted, and are summarized in Figure 4.

### 2.4. Effect of BAL on Markers of Activated Astrocytes (GFAP)

**Figure 5:**
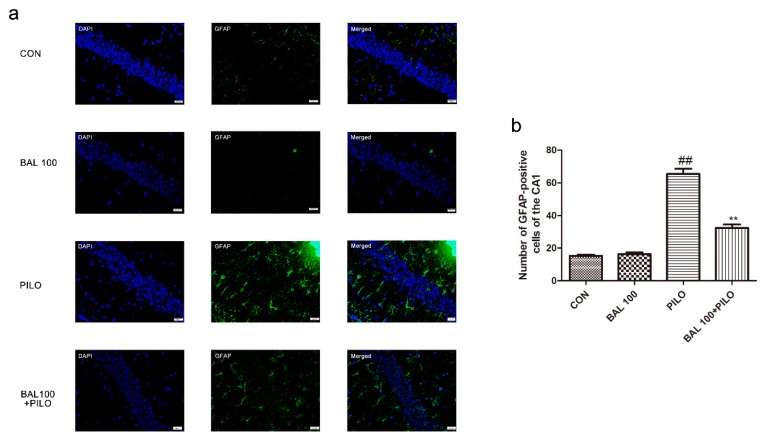
Effect of BAL on astrocyte activation in the hippocampus at 72 h after convulsion are shown with high power (×400 magnification). GFAP-positive cells in CA_1_ are shown for CON group, PILO group, and BAL 100 group, BAL 100 + PILO group (**a**). Bar: 20 μm. Quantitative analysis of GFAP-positive cells in the hippocampal CA_1_ (**b**). Results are expressed as mean ± SEM. ^##^ *p* < 0.05 (Student-Newman-Keuls) as compared to control group, ** *p* < 0.01 (Student-Newman-Keuls) as compared to the PILO group (*n* = 6, One-way ANOVA and the Student-Newman-Keuls).

Few glial fibrillary acidic protein (GFAP) positive cells were found in the hippocampal CA_1_ region of the CON group. At 72 h after SE, the number of GFAP-positive cells significantly increased in the hippocampus in PILO group compared with that in the CON group (*p* < 0.01). The mice pretreated with BAL (100 mg/kg) had a significantly reduced number of GFAP-positive cells (*p* < 0.01). The number of GFAP-positive cells was quantitatively analyzed in the CON, PILO, and BAL 100 groups and was statistically shown in the histogram. The results are summarized in Figure 5.

### 2.5. BAL Restores GABAergic Neurotransmission and Inhibits Neuroinflammation

**Figure 6:**
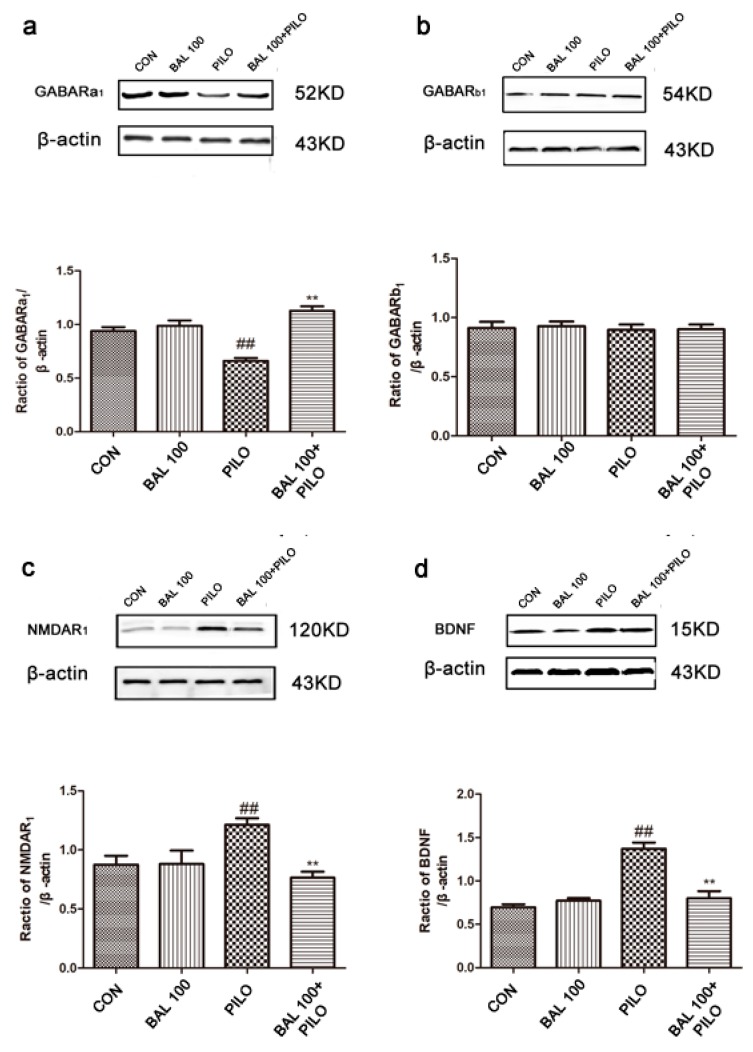

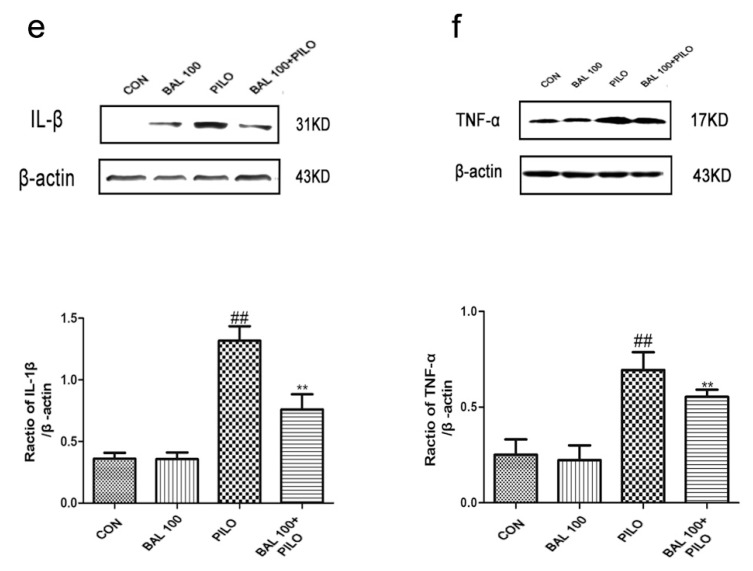
The expression of GABARa_1_ (**a**), GABARb_1_ (**b**), NMDAR_1_ (**c**), d: BDNF (**d**), IL-1β (**e**) and TNF-α (**f**) in brain tissue. Immunoblots showed target protein levels (**a**–**f**) 72 h after PILO-induced seizures. Data were expressed as mean ± SEM (*n* = 6), ^##^ *p* < 0.01 (Student-Newman-Keuls) compared to control group, ** *p* < 0.05 (Student-Newman-Keuls) compared to PILO group (One-way ANOVA and the Student-Newman-Keuls).

The cerebral levels of NMDAR_1_, TNF-α, IL-1β, and BDNF increased significantly after PILO administration (*p* < 0.05) and were restored in the BAL-treated (100 mg/kg) mice 72 h after the seizure onset (*p* < 0.01). Furthermore, high-dose BAL significantly upregulated GABAR_a1_ compared to the untreated epileptic mice (*p* < 0.05) but did not affect the levels of GABAR_b1_. The results are summarized in Figure 6.

## 3. Discussion

Valerian plant is used to treat insomnia, mood disorders, anxiety, menstrual cramps, and psychological stress. BAL is derived from the extracts of the rhizomes and root of the valerian plant. We found that BAL significantly attenuated the in the PILO-injected murine model. None of the mice in the highest dose group died. It delayed the latency of the first seizure and the onset of SE, decreased the incidence of SE, and exerted an anti-convulsive effect by alleviating PILO-induced abnormal EEG patterns. VPA, a common anti-seizure drug [26], was used as the positive control and mitigated the behavioral, electrographic and histopathological changes induced by PILO, as previously described.

Astrocytes are known to participate in the immune response [27,28], and in the regulation of ion homeostasis [28], as well as in the control of the concentration of various neurotransmitters, including GABA [29] and Glu [30]. Due to these important functions, astrocytes are an interesting target for the understanding of changes before, during, and after a seizure, as well as the mechanism that permits the development of a SE [24,31].

Activated astrocytes are one of the most important pathological changes in epilepsy. Reactive gliosis, a component of neuroinflammation that involves structural and metabolic changes in astrocytes and microglia, is often a prominent feature of mesial temporal lobe human epilepsy and most animal models of recurrent seizures [32]. The major mechanisms by which glia can facilitate the development of seizures and epilepsy include increased excitability and inflammation [33].

Activated astrocytes affect the balance of glutamatergic and GABAergic nerve in three ways. First, activated astrocytes release Glu [34]. Studies have shown that hypoosmotic medium caused glutamic acid release from astrocytes in culture, a process that was prevented by several volume-sensitive anion channel blockers. They concluded that cell swelling occurring during ischemia or seizures could cause astrocytic glutamic acid release [32]. Second, there are two kinds of glutamate transporter expressed in astroglial cells and primarily astrocytes. Activated astrocyte cells also affect glutamate transporter, and the transport capacity is decreased [32]. In TLE, reduced expression of the astroglial glutamate transporters was found [35]. Third, activated astrocytes cells release inflammatory cytokines [36]. Cytokines are usually expressed at low levels in the healthy brain and show a considerable increase in the epileptic brains [33]. In fact, neuronal excitability is often characterized by the accumulation of pro-inflammatory cytokines, which further influence synaptic transmission and can exacerbate seizures. For instance, TNF-α and IL-1β have been directly associated with the occurrence of seizures [35,37]. These inflammatory cytokines are secreted in the epileptic brain by astrocytes which are activated and proliferate rapidly in neuropathological conditions [37]. The cytokines aggravate astrogliosis and enhance the epileptogenic inflammatory signaling by accumulating at synapses and activating specific receptors [38], thus triggering a vicious cycle which culminates in neuronal excitability and seizures [39,40]. Furthermore, the pro-inflammatory cytokines reduce the seizure threshold, resulting in chronic neuronal hyper-excitability and spontaneous recurrent seizures [41]. IL-1β also directly increases neuronal excitability by inhibiting Ca^2+^ channel signals [42] and reducing GABA_a_ receptor-mediated response [40,43]. Elevated TNF-α levels also decrease inhibitory transmission. Exposure of mature rat and mouse hippocampal neurons to TNF-α induced a rapid and persistent decrease in inhibitory synaptic strength and downregulated GABA_a_ receptor levels [44].

Activated astrocytes affect not only glutamic acid and GABA secretion but also NMDA receptors. Astrocytes regulate the expression of the neuronal N-methyl-D-aspartate receptor (NMDAR) subunits 2A and 2B [45]. A study carried out on a mice model of pilocarpine-induced SE in which gliotransmitter release was genetically inhibited, showed that reduction in surface expression and function of neuronal NMDA receptors can delay seizure onset and attenuate the subsequent progressive increase in seizure frequency, suggesting that astrocytes may be important in modulating epileptogenesis [46].

Brain-derived neurotrophic factor (BDNF) is a neurotrophic factor that induces epilepsy both directly, as well as indirectly through the GABAergic and glutamatergic neurons [47]. The pilocarpine-induced model of SE showed increased BDNF expression in the brain [48]. Furthermore, acute administration of BDNF into the CA_3_ of the hippocampus, dentate gyrus, and medial entorhinal cortex resulted in neuronal hyper-excitability [49]. BDNF also increased NMDA signals in TLE patients and attenuated inhibition of GABAergic postsynaptic cells by downregulating Cl^−^ transport [50].

Taken together, astrocytes in epilepsy brain lose their original function of maintaining brain homeostasis, resulting in dysfunction of excitatory and inhibitory neurotransmitters in the brain, thus causing epilepsy.

In experimental animal models of epilepsy, astrocytes are rapidly activated with the hypertrophy of cell bodies, thus increasing the expression of GFAP [51]. The present study showed that BAL administration to PILO treated animals reduced the number of GFAP-positive cells and minimized morphological changes, indicating that BAL attenuated the seizure-induced activation of astrocytes. After administration PILO, compared with PILO group, GFAP fluorescence signal decreased, GABA and GABARa expression increased, TNF-α, ILβ-1, BDNF, and NMDAR_1_ expression decreased. The results showed that BAL decreased astrocyte activation, decreased the secretion of inflammatory cytokines in brain tissue, and improved the imbalance between excitability and inhibition in the brain.

The PILO-induced epilepsy model has helped to elucidate the inflammatory mechanisms underlying epileptogenesis [52]. There is evidence that PILO directly activates the cholinergic system to trigger seizures, although a recent study raised the possibility of an indirect role via peripheral inflammation, which broke the blood-brain barrier (BBB) prior to the onset of SE [53]. Furthermore, pre-treatment with atropine, a muscarinic antagonist, counteracted the cholinergic effects of systemically injected PILO, indicating that cholinergic neuron activation is the sole factor triggering PILO-induced SE [54].

Seizure-injured neurons, known as interneurons found in the hippocampus, are patho-physiologically related to recurrent seizures [55]. The limbic brain region constituted by the cortex and hippocampus plays a vital role in epileptogenesis and its associated co-morbidities, including learning and memory deficits [55]. The CA_1_, CA_3_ and dentate gyrus of the hippocampus, amygdala, and cortex are vulnerable to neuronal loss in TLE [56,57]. Nissl staining of the hippocampal CA_1_ and CA_3_ regions showed significant neurodegeneration, with reduced Nissl bodies, disintegrated cell membrane and pyknosis of nucleolus in 72 h after a seizure. BAL pre-treatment ameliorated these effects and restored the number of Nissl bodies and morphological features. Therefore, BAL protects against PILO-induced neurodegeneration.

## 4. Materials and Methods

### 4.1. Establishment of the Murine Epileptic Model

Adult male ICR (Institute of Cancer Research) mice (aged five weeks and weighing 21–25 g) were obtained from the Experimental Animal Center of Ningxia Medical University. All animals were housed in standard plastic cages under specific pathogen-free environment under controlled temperature (23 ± 2 °C) and relative humidity (60 ± 10%), and 12 h light/dark cycle with free access to food and water. The mice were treated humanely, and the experiments were conducted in accordance with the National Institute of Health Guidelines for the Care and Use of Laboratory Animals and approved by the Animals Ethics Committee of Ningxia Medical University (certificate number of SYXK Ningxia 2015-001). Convulsion model of mice was induced by an intraperitoneal injection of PILO (280 mg/kg Sigma, USA) and atropine sulfate hydrate (1 mg/kg Shanghai Yuanye Bio-technology Co. Ltd., Shanghai, China) 15min before injecting PILO [54].

The mice were observed continuously for 60 min for any behavior indicative of seizures, and graded according to a modified version of the Racine scale [58]. SE incidence, mortality rate and convulsion onset time were also recorded. Convulsion was defined as the occurrence of grade 4–5 seizures based on the Racine scale. SE was defined as a phase of continuous seizures that lasted for at least 5 min or seizures that recurred at extremely short intervals (<1 min) and thus established a persisting epileptiform condition [59,60]. When the mice experienced grade 4–5 seizures or SE for 60 min, the convulsions were terminated by an intraperitoneal injection of diazepam (1 mg/kg) to reduce mortality [54]. During the convulsive period, all mice were housed in an incubator and fed with egg mash until they resumed eating normal pellets.

### 4.2. Treatment Regimen

The mice were randomly divided into the following seven groups (*n* = 24 each):

- CON—2% CMCNa
- BAL—100 mg/kg BAL
- PILO—2% CMCNa + PILO
- VPA 200—200 mg/kg VPA + PILO
- BAL 25 + PILO—25 mg/kg BAL + PILO
- BAL 50 + PILO—50 mg/kg BAL + PILO
- BAL 100 + PILO—100 mg/kg BAL + PILO

Thirty minutes before PILO injection, the mice were intraperitoneally injected with BAL (dissolved in 2% CMCNa, Shanghai Yuanye Bio-technology Co. Ltd., LOT Z24N8S49019, purity > 98% by HPLC), 2% CMCNa or valproate sodium (VPA). BAL was sonicated for 15 min before injection to ensure homogeneity.

### 4.3. Cortical Electro-Encephalograph (EEG) Recording

Ten mice per group were anesthetized by 350 mg/kg chloral hydrate and fixed onto the stereotaxic apparatus. Polyurethane-coated stainless steel monopolar recording and reference electrodes (100 μm diameter) were surgically implanted into the left frontal cortex (anterior–posterior coordinate relative to bregma AP = −0.5 mm, mediolateral coordinate relative to midline ML = 1.5 mm) and the right occipital cortex (AP = −3.5 mm, ML = 2.0 mm) respectively [54]. Dental auto-polymerized acrylic was used to fix the electrodes. The mice were placed in an incubator, fed with soaked rodent food, and allowed to recover for five days before recording. The mice were carefully placed into individual plexiglass boxes, and the electrodes were linked to a biological signal processing system (SMUP-U4) to detect EEG seizures for 60 min after PILO injection. The mice were allowed to be to move freely in the boxes for least 15 min to acclimatize them. BAL, VPA, and CMCNa were injected 30 min before PILO, and EEG recordings were started 2 min after PILO injection. The results were amplified 800 times and filtered between 1 and 50 Hz. All data were stored on a hard disk and subjected to fast Fourier power spectral analysis.

### 4.4. Histopathological Evaluation

Seventy-two hours after PILO injection, the mice were deeply anesthetized (350 mg/kg chloral hydrate, i.p.) and trans-cardially perfused with 0.9% saline solution followed by Bouin’s fixative (saturated picric acid, methyl alcohol, and glacial acetic acid at the ratio of (25:5:1) [60]. The brain was quickly removed and fixed in Bouin’s fixative for 12 h at 4 °C. After washing with purified water for 5 min and dehydrating with 30% cane sugar solution, the brain tissues were embedded into OTC (optimal cutting temperature compound), and 10 µm coronal sections were cut. The sections were then subjected to Nissl staining [61]. After washing with PBS, the brain sections were immersed in 0.9% cresyl violet for 1 h at 56 °C, rinsed quickly in purified water, differentiated in 95% ethanol for 5 s, cleared in xylene for 30 s, and mounted with neutral balsam. The number of surviving hippocampal CA_1_ and CA_3_ pyramidal cells per 1 mm was determined in each section at 400× magnification by two investigators blinded to the samples [54].

Glial fibrillary acidic protein is an astrocyte-specific cytoskeletal protein used as a reliable marker for reactive astrogliosis [62]. Immunofluorescence for brain tissue was performed on frozen sections, which were prepared to use the same method for Nissl staining. The sections were subsequently washed with PBS and incubated in normal goat serum (Tianjing TBD Biotechnology, Tianjin, China) for 60 min, followed by incubation at 4 °C overnight with the primary mouse anti-GFAP antibody (1:200). The following day, the brain sections were rinsed with PBS to remove the unbound antibodies and then incubated with FITC-labeled goat anti-rabbit IgG for 2 h, followed by 4′,6-diamidino-2-phenylindole for 5 min at room temperature. Regardless of the intensity of labeling, all GFAP-positive cells were counted by two investigators blinded to the classification of tissues under a ×40 objective lens in the hippocampal CA_1_ region of each section in the predefined areas via laser scanning confocal microscopy.

### 4.5. Enzyme-Linked Immunosorbent Assay (ELISA)

After sacrificing the mice 60 min post-PILO or CMCNa injection, the whole brains were collected, weighed, and homogenized. The protein content was determined by BCA method. The supernatants of GABA (Nangjing Jiancheng Bio, Nanjing, China) and GUL (Nangjing Jiancheng Bio) were evaluated by ELISA kits according to the manufacturer’s protocol.

### 4.6. Western Blotting

The brains were removed 72 h after convulsion and rapidly homogenized in ice-cold lysis buffer (Nanjing Key Gen Biotech Co., Nanjing, China). After centrifuging at 15,000g and 4 °C for 15 min, the supernatant was collected and quantified by the BCA method. Equal amounts of protein per sample were resolved by SDS-PAGE and transferred electrophoretically onto PVDF membranes. The latter was blocked with 5% non-fat milk, and incubated overnight with primary antibodies (Proteintech Group) against GABAR_a1_ (1:1000), GABAR_b1_ (1:1000), NMDAR_1_ (1:1200), BDNF (1:1000), β-actin (1:1000), TNF-α(1:400) and IL-1(1:500) at 4 °C. The blots were washed thrice and incubated with horseradish peroxidase-linked anti-rabbit IgG (1:2000; Proteintech Group) at room temperature for 2 h and developed using chemiluminescence (Westernbright^TM^ ELC Lot No: 170915-11). The density of each band was quantified by the Quantity One software (Bio-Rad Laboratories, Hercules, CA, USA).

### 4.7. Statistical Analysis

Results were expressed as mean -SEM. One-way ANOVA and the Student–Newman–Keuls test were employed for post hoc comparisons to determine the differences between the control and experimental groups. Student’s t-test was performed for paired samples. Statistical significance was set at *p* < 0.05.

## 5. Conclusions

To summarize, BAL exhibited dose-dependent anti-convulsion effects in a mouse model of PILO-induced SE. In terms of mechanism, it may be mediated by reducing the inflammatory response in the PILO-induced brain and restoring the balance of GABAergic and glutamatergic neurons. Thus, BAL is a promising adjuvant drug for treating epilepsy. Further studies are needed to dissect the neuro-molecular mechanisms of BAL action.

## Figures and Tables

**Table 1:** Effect of baldrinal (BAL) on pilocarpine (PILO)-induced convulsions and lethality. Result for latency to the first convulsion was expressed as mean ± SEM (*n* = 12). Data on survivors and the number of animals with with status epilepticus (SE) was calculated as percentages.

Groups	Number of Animals/Groups	Latency to First Convulsion (second)	Percentage Convulsion (%)	Percentage SE (%)	Percentage of Survival (%)
CON	12	No	0	0	100
BAL 100	12	No	0	0	100
PILO	12	544 ± 42 ^##^	100	100 ^a^	66.7(8/12)
VPA 200	12	No	0	0	100
BAL 25 + PILO	12	587 ± 55	100	100(12/12) ^b^	66.7(8/12)
BAL 50 + PILO	12	1178 ± 97 *	100	75(9/12) ^b^	91.67(11/12)
BAL 100 + PILO	12	3476 ± 48 **	41.6(5/12)	0 ^b^	100 ^b^

## *p* < 0.01 as compared to the control group; *p < 0.05 as compared to the PILO group; **p < 0.01 as compared to the PILO group (One-way ANOVA and the Student-Newman-Keuls) ^a^ *p* < 0.01 as compared to the PILO group ^b^ *p* < 0.05 as compared to the PILO group (χ^2^ method and Fischer’s exact probability test).
